# Relation between the frequency of CD34^+ ^bone marrow derived circulating progenitor cells and the number of diseased coronary arteries in patients with myocardial ischemia and diabetes

**DOI:** 10.1186/1475-2840-10-107

**Published:** 2011-11-25

**Authors:** Ilkay Bozdag-Turan, R Goekmen Turan, C Hakan Turan, Sophie Ludovicy, Ibrahim Akin, Stephan Kische, Nicole S Arsoy, Henrik Schneider, Jasmin Ortak, Tim Rehders, Tina Hermann, Liliya Paranskaya, Peter Kohlschein, Manuela Bastian, A Tulga Ulus, Kurtulus Sahin, Hueseyin Ince, Christoph A Nienaber

**Affiliations:** 1 Department of Internal Medicine, Division of Cardiology, University Hospital Rostock, Germany; 2 Institute of Clinical Chemistry & Laboratory Medicine, University of Rostock, Germany; 3 Turkiye Yuksek Ihtisas Hospital, Department of Cardiovascular Surgery, Ankara, Turkey; 4 Institute for Clinical Research and Statistics, Cologne, Germany

**Keywords:** CD34/45^+^, ischemic heart disease, diabetes, frequency

## Abstract

**Background:**

Bone marrow-derived circulating progenitor cells (BM-CPCs) in patients with coronary heart disease are impaired with respect to number and mobilization. However, it is unknown whether the mobilization of BM-CPCs depends on the number of diseased coronary arteries. Therefore, in our study, we analysed the correlation between the diseased coronary arteries and the frequency of CD34/45+ BM-CPCs in peripheral blood (PB) in patients with ischemic heart disease (IHD).

**Methods:**

The frequency of CD34/45^+ ^BM-CPCs was measured by flow cytometry in 120 patients with coronary 1 vessel (IHD1, n = 40), coronary 2 vessel (IHD2, n = 40), coronary 3 vessel disease (IHD3, n = 40) and in a control group of healthy subjects (n = 40). There was no significant difference of the total number of cardiovascular risk factors between IHD groups, beside diabetes mellitus (DM), which was significantly higher in IHD3 group compared to IHD2 and IHD1 groups.

**Results:**

The frequency of CD34/45^+ ^BM-CPCs was significantly reduced in patients with IHD compared to the control group (CD34/45^+^; p < 0.001). The frequency of BM-CPCs was impaired in patients with IHD3 compared to IHD1 (CD34/45^+^; p < 0.001) and to IHD2 (CD34/45^+^; p = 0.001). But there was no significant difference in frequency of BM-CPCs between the patients with IHD2 and IHD1 (CD34/45^+^; p = 0.28). In a subgroup we observed a significant negative correlation between levels of hemoglobin AIc (HbAIc) and the frequency of BM-CPCs (CD34/45^+^; p < 0.001, r = -0.8).

**Conclusions:**

The frequency of CD34/45^+ ^BM-CPCs in PB is impaired in patients with IHD. This impairment may augment with an increased number of diseased coronary arteries. Moreover, the frequency of CD34/45^+ ^BM-CPCs in ischemic tissue is further impaired by diabetes in patients with IHD.

## Introduction

Circulating progenitor cells are primitive bone marrow (BM) cells that have the capacity to proliferate, migrate and differentiate into various mature cell types [1,2]. These bone marrow-derived circulating progenitor cells (BM-CPCs) express unique surface markers, such as CD34+ and the early hematopoietic cell marker CD133+ (AC133+). During ischaemia, populations of BM-CPCs are mobilized and recruited to ischaemic areas, accelerating the neovascularization process [3]. Previous studies demonstrate that cardiovascular risk factors (CVRFs) for coronary artery disease correlate with a reduced number and functional activity of circulating endothelial progenitor cells [4]. Moreover, diabetic patients showed impaired proangiogenic and colony-forming activity of circulating progenitor cells [5,6]. However, it is unknown whether the mobilization of BM-CPCs relates to the number of diseased coronary arteries in patients with IHD. In this study, we analysed the frequency of CD34/45^+ ^BM-CPCs and their relationship with the number of diseased coronary arteries in patients with ischaemic heart disease (IHD).

## Materials and methods

### Study Protocol and Study Population

The study included 120 IHD patients and 40 healthy subjects between 18-80 years of age. We selected a control group of 40 healthy subjects without overt heart disease and/or major cardiovascular risk factors (diabetes, smoking, hypertension, hypercholesterolemia, and familial history). A cardiovascular risk factors (CVRFs) score including age > 40 years, male sex, hypertension, diabetes, smoking, positive family history and hypercholesterolemia was calculated according to Vita et al. [7] Hypertension was defined as a history of hypertension for > 1 year that required the initiation of antihypertensive therapy by the primary physician. Smoking was defined as patients revealing a history of smoking for > two pack-years and current smoking. Positive family history was defined as documented evidence of coronary artery disease (CAD) in a parent or sibling before 60 years of age. Hypercholesterolemia was defined as fasting low-density-lipoprotein (LDL) cholesterol levels exceeding 130 mg/dl. Diabetes was defined as the need for oral antidiabetic drug therapy or insulin use.

Exclusion criteria were the presence of acutely decompensated heart failure with a New York Heart Association (NYHA) class of IV, infectious or inflammatory disease, active bleeding, surgery or trauma within two months, renal or liver dysfunction, thrombocytopenia, or anaemia, a severe comorbidity and alcohol or drug dependency, a history of other severe chronic diseases or cancer, or unwillingness to participate. The study conforms with the principles outlined in the Declaration of Helsinki and was approved by the local ethics committee. Written consent was obtained from each patient.

### Coronary Angiography and Left Ventriculography

All IHD patients underwent left heart catheterization, left ventriculography and coronary angiography. Cardiac catheterization was performed according to the guidelines for coronary angiography of the American College of Cardiology and the American Heart Association [8]. Cardiac function was determined by left ventriculography. Cardiac function was evaluated by global EF. Global Ejection Fraction (EF) was measured with Quantcor software (Siemens, Erlangen/Germany). The extent of coronary artery disease was scored by at least two independent interventional cardiologists as 0 (stenosis < 50 percent), 1 (stenosis of any main coronary artery > 50 percent), 2 (stenosis of two main coronary arteries > 50 percent), and 3 (stenosis of three main coronary arteries > 50 percent).

### Frequency of CD34/45^+ ^BM-CPCs

10 ml peripheral blood (PB) were taken from the study population to measure of BM-CPCs as well as conduct biochemical analyses. CD34/45^+ ^BM-CPCs were quantified by flow cytometry (EPICS-XL, Beckmann Coulter) in 5 of 10 ml PB from each subject. Assessments in IHD patients (n = 120) were done during cardiac catheterization. For the control group (n = 40), measurements of CD34/45^+ ^were performed on day 1 of admission. PB samples were analysed within two hours.

Samples were stained with fluorescein isothiacyanate (FITC) conjugate of a CD45^+ ^antibody (clone J33, Coulter/Immunotech, Marseille/France) that detects all isoforms and glycoforms of the CD45 family, phycoerythrin (PE) conjugate of a CD34^+ ^antibody (clone 581, Coulter/Immunotech, Marseille/France) that detects a class III epitope on all glycoforms of the CD34^+ ^antigen. Control samples were stained with CD45^+ ^FITC and an IgG1 PE (Coulter/Immunotech, Marseille/France) isotype.

For each patient, EDTA blood samples were labeled with CD34/45^+ ^and IgG1/CD45. All tubes were incubated at room temperature in the dark. After incubation, mainly red blood cells were lysed with ammonium chloride and washed with phosphate-buffered saline (PBS). Samples were then stored on ice at 4°C in the dark for 20 min and analysed by flow cytometry [9,10].

Samples were subjected to a 2D side scatter-fluorescence dot plot analysis. After appropriate gating, the concentration of BM-CPCs with low cytoplasmic granularity (low side ward scatter) was quantified and expressed as a concentration of cells per million white blood cells.

### Biochemical Measurements

Serum creatine phosphokinase (CPK) values (normal range: 24 - 195 U/l), inflammatory markers such as C - reactive protein (CRP) (normal range < 0.5 mg/dl) leukocytes (normal range: 4 - 12 × 10^3^/μl) and routine laboratory tests with HbA1c were measured in the remaining 5 of 10 ml PB from each study subject.

### Statistical Analysis

Continuous data are presented as mean ± SD. A comparison of the distributions of a continuous variable between two independent groups was performed using the two-sided nonparametric Mann-Whitney test. The type I error rate α was chosen as 5% and two-sided p-values equal or less than 0.05 were interpreted as statistically significant. Some qualitative baseline characteristics were compared using the Fisher's Exact-Test.

A bivariate regression analysis was presented in a graphical form and Pearson's correlation coefficient was obtained.

Statistical significance was accepted, if the corresponding two-sided p-value was smaller or equal to 0.05. Statistical analysis was performed with SPSS for Windows (Version 15.0).

## Results

### Baseline characteristics of the patients and healthy control subjects

We included 120 patients with IHD and 40 healthy subjects without heart disease in the study. The baseline characteristics of the study population are depicted in table 1. Interestingly, there was a significant difference in the number of DM patients in the IHD3 group compared to groups IHD1 and IHD2. In contrast, there was no significant difference in the number DM patients between IHD1 and IHD2. Also, no significant differences were observed in other baseline characteristics and demographics of patients between all IHD groups (table 1).

**Table 1:** Baseline clinical characterics of the study population.

	IHD1 (n = 40)	IHD 2 (n = 40)	IHD 3 (n = 40)	P	Control Group (n = 40)
Age	60 ± 15	60 ± 11	64 ± 15	NS	64 ± 10
Male	24	26	25	NS	21
Cardiovascular Risk Factors % (n)					
Hypertension	80 (n = 32)	80 (n = 32)	78 (n = 31)	NS	-
Hyperlipidemia	60 (n = 24)	60 (n = 24)	53 (n = 21)	NS	-
Smoking	68 (n = 27)	75 (n = 30)	63 (n = 25)	NS	-
Positive family history of CAD	40 (16)	40 (n = 16)	35 (n = 14)	NS	-
Diabetes	20 (n = 8)	20 (n = 8)	60 (n = 24)	p = 0.001	-
OHA	15 (n = 6)	15 (n = 6)	45 (n = 18)	NS	-
Insulin	5 (n = 2)	5 (n = 2)	15 (n = 6)	NS	-
Total number of CVRFs	4.2 ± 0.8	4.3 ± 0.8	4.4 ± 0.9	NS	-
Infarct-related vessel (LAD/LCX/RCA)	19/8/13	20/10/11	20/8/12	NS	-
PTCA/Stent at the time of AMI	40/40	40/40	40/40	NS	-
Ejection fraction (%)	45 ± 10	43 ± 11	40 ± 10	NS	69 ± 9
Medication (%)					-
Aspirin	100	100	100	NS	-
Clopidogrel	100	100	100	NS	-
ACE inhibitor or AT II blocker	100	100	100	NS	-
Beta-blocker	100	100	100	NS	-
Aldosterone Antagonist	25	25	30	NS	-
Statin	100	100	100	NS	-

Ischemic heart disease (IHD), Oral hypoglycaemic agent (OHA), Coronary artery disease (CAD), Percutaneous transluminal coronary angioplasty (PTCA), Left anterior descending coronary artery (LAD), Left circumflex artery (LCX), Right coronary artery (RCA), Coronary artery disease (CAD), Cardiovascular risk factors (CVRFs), Acute myocardial infarction (AMI), None significant (NS).

### Frequency of CD34/45^+ ^BM-CPCs

**Figure 1:**
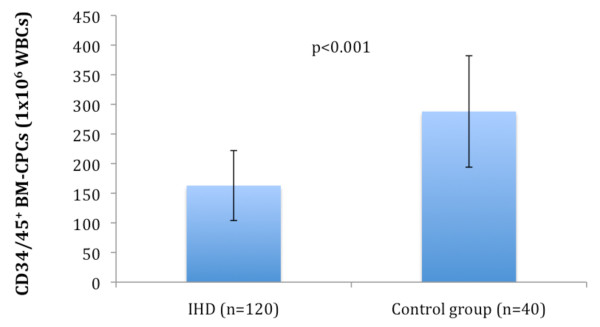
**The frequency of CD34/45^+ ^BM-CPCs were significanty reduced a total of IHD patients as compared to healthy subjects**.

**Figure 2:**
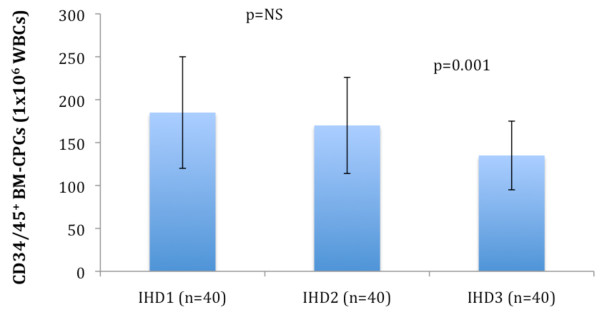
**The frequency of CD34/45^+ ^BM-CPCs was impaired in patients with IHD3 as compared to IHD1**. But there was no significant difference in frequency of BM-CPCs between the patients with IHD2 and IHD1.

The frequency of CD34/45^+ ^BM-CPCs was measured by flow cytometry in 120 IHD patients as well as in 40 healthy subjects. The frequency of CD34/45^+ ^BM-CPCs was significantly reduced in IHD patients (n = 120) compared to healthy subjects (n = 40) (CD34/45^+^; p < 0.001) (Figure 1). We observed a significant decrease of CD34/45^+ ^frequency in patients with IHD3 (n = 40) compared to IHD1 (n = 40) (CD34/45^+^; p < 0.001) and IHD2 (n = 40) (CD34/45^+^; p = 0.001) patients. There was no significant difference of CD34/45^+ ^frequency between IHD1 and IHD2 patients (CD34/45^+^; p = 0.28) (Figure 2). Additionally, the number of leukocytes by FACS-analysis as well as by biochemical analysis in each group were measured to exclude a negative effect of leukocytes on frequency of CD34/45^+ ^BM-CPCs. There was no significant difference in the number of leukocytes between each group (IHD1; 8 ± 2 × 10^3^/μl, IHD2; 8 ± 3 × 10^3^/μl, 8 ± 2 × 10^3^/μl, p = NS)

### Relationship between DM and frequency of BM-CPCs in IHD patients

**Figure 3:**
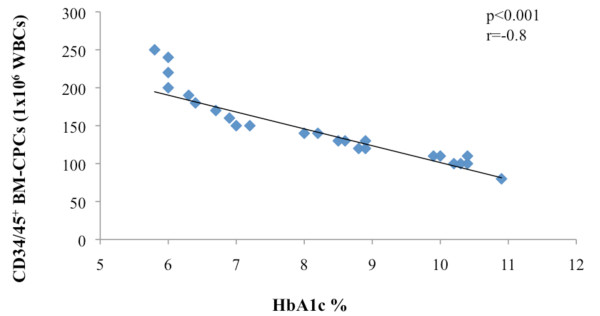
**We observed a significantly inverse correlation between the frequency of CD34/45^+ ^BM-CPCs and levels of HbA1C in total of IHD patients with diabetes (n = 40)**.

**Figure 4:**
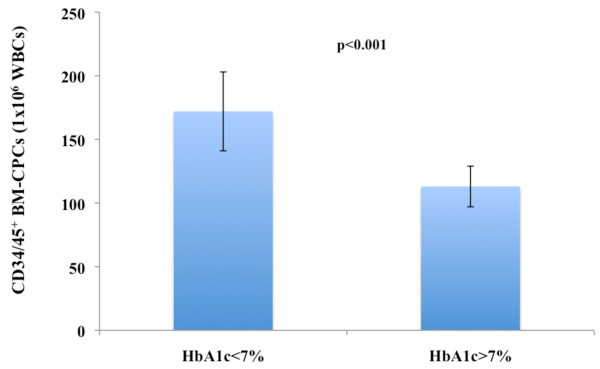
**The CD34/45^+ ^BM-CPCs frequency was significanty reduced in a total of DM patients with HbA1C > 7% (n = 20) as compared to DM patients with HbA1C < 7% (n = 20)**.

After we observed that patients with IHD3 had a significant higher incidence of DM compared to IHD2 and IHD1, we investigated the link between a DM and the frequency of BM-CPCs in IHD patients in a subgroup of patients. We observed a significant negative correlation between the levels of HbA1c and the frequency of CD34/45^+ ^(p < 0.001 r = -0.8, n = 40) (Figure 3). Moreover, the BM-CPCs frequency was significantly reduced in DM patients with HbA1c > 7% (n = 20) compared to DM patients with HbA1c < 7% (n = 20) (CD34/45^+^; p < 0.001) (Figure 4).

## Discussion

In this controlled study we examined the correlation between the diseased coronary arteries and the frequency of CD34/45+ BM-CPCs in patients with IHD.

Coronary artery disease results from a chronic inflammatory disease of the vascular wall and leads to vessel occlusion and organ damage [11]. Despite intense efforts to determine the pathogenesis of atherosclerosis, this process remains poorly understood. Reports suggest that risk factors and a genetic predisposition together induce inflammatory processes that leads to cell damage and impairs regeneration within the vessel wall [12,13]. Since resident endothelial cells infrequently proliferate, [14] it has been postulated that there are other sources of vascular replenishment in response to continuous damage [15]. Circulating progenitor cells derived from bone marrow circulate in the peripheral blood and have been implicated in neoangiogenesis after tissue ischemia has occurred [16-19]. BM-CPCs are capable of proliferating and differentiating into endothelial cells and are therefore ideal candidates for vascular regeneration [20,21]. Experiments in animals show that the systemic application or mobilization of stem cells and progenitor cells beneficially influences the repair of endothelial cells after injury and the progression of atherosclerosis [22-28]. Clinical trials indicated a beneficial effect of intracoronary infusion of BMCs, or circulating progenitor cells (CPCs), on myocardial function in patients with acute myocardial infarction (AMI) [29,30]. Previous studies demonstrated, that the mobilization and functional activity of CD34/45^+ ^and CD133/45^+ ^BM-CPCs significantly increased after an intracoronary infusion of BMCs in patients with ischemic heart disease [31,32]. BM-CPCs can also be used as a predictive biomarker for cardiovascular risk, vascular function and the extent of cardiac repair [33]. Moreover, smoking is associated with depletion of CD34+ and CD133+ endothelial progenitor cells in patients with coronary artery disease [34]. In a large clinical study, Hill et al [35] reported that high-risk individuals have fewer BM-CPCs compared with to their low-risk counterparts. In contrast, Werner et al [11] identified a significant association between increased numbers of BM-CPCs and a decreased risk of major cardiovascular events and hospitalization in patients with coronary artery disease. BM-CPCs mobilization can also predict severe endothelial dysfunction in patients with coronary heart disease [36]. Moreover, the transient increase in CD34/45^+ ^and CD133/45^+ ^BM-CPCs reached a maximum after three weeks of regular symptom-limited (ischemic and/or subischemic) exercise training, but did not persist until 3 months after the regular training after acute myocardial infarction [37,38]. However, it is unknown whether the frequency of CD34^+ ^BM-CPCs relates to the number of diseased coronary arteries in patients with IHD. We demonstrated in our study that the frequency of BM-CPCs was significantly impaired in patients with IHD3 compared to IHD2 and IHD1. In patients with heart failure and preserved LVEF, diabetes is associated with a significantly increased risk of developing adverse HF outcomes [39]. Diabetes mellitus is associated with both an increased risk of atherosclerotic disease and poor outcomes after vascular occlusion. The clinical severity of vascular occlusive disease in diabetics has in part been attributed to impaired collateral vessel development [40] Extensive studies have shown that the numbers of circulating angiogenic cells are significantly lower in type II diabetes and their angiogenic potential is also dramatically diminished. These cells display defective adhesion to the endothelium, reduce proliferation rate, and impaire ability to create new vascular structures [41,42]. Patients with type 1 diabetes have reduced levels of endothelial progenitor cells and their functional capacity is impaired. Reduced nitric oxide bioavailability and increased oxidative stress play a role in endothelial progenitor cell dysfunction in these patients. Similarly, insulin resistance impairs circulating angiogenic progenitor cell function. On the other hand, increases in fractalkine level and the number and functional changes of blood dendritic cells might contribute to diabetic coronary atherosclerosis and plaque destabilization [43-45]. On the basis of these findings, it is tempting to speculate that the decreased frequency of CD34^+ ^BM-CPCs by DM lead to a progression of atherosclerosis and increase the number of diseased coronary arteries in patients with IHD. In line with this hypothesis we observed in our study a significant higher incidence of DM in patients with IHD3 compared to IHD2 and IHD1. Furthermore, we demonstrated that the frequency of BM-CPCs negatively correlated with the level of HbA1c in IHD patients with DM. The CD34^+ ^BM-CPCs frequency in DM patients with HbA1c > 7% was significantly reduced compared to DM patients with HbA1c < 7%. Recent studies have shown that the PPARϒ agonist pioglitazone treatment increases the number and function of BM-CPCs in type 2 DM patients with coronary artery disease [46,47]. Improved levels of HbA1c by pharmacological therapy may lead to an increase of BM-CPCs frequency and functional activity and thereby may enhance the vascular regeneration in IHD patients with DM.

In the present study we could demonstrate that CD34^+ ^BM-CPCs frequency was impaired in patients with IHD. This impairment correlates with an increase in the number of diseased coronary arteries. Moreover, the frequency of CD34^+ ^BM-CPCs is further impaired by DM in patients with IHD.

## Abbreviations

BM-CPCs: Bone marrow-derived circulating progenitor cells; IHD: Ischemic heart disease; PB: Peripheral blood; DM: Diabetes mellitus; HbAIc: Hemoglobin AIc; CVRFs: Cardiovascular risk factors; CAD: Coronary artery disease; LDL: Low-density-lipoprotein; NYHA: New York Heart Association; EF: Ejection Fraction; CPK: Creatine phosphokinase; CRP: C - reactive protein; AMI: Acute myocardial infarction; FITC: Fluorescein isothiacyanate; PE: Phycoerythrin; PBS: Phosphate-buffered saline.

## Competing interests

The authors declare that they have no competing interests.

## Authors' contributions

IBT, RGT, CHT and SL conceived the study, arranged the collaboration, initiated the manuscript, edited and compiled the final version for submission. IA, SK, NSA, HS, JO, TR, LP, HI an CAN conceived of the study, and participated in its design and coordination. PK and MB performed Laboratory analyses and study design. ATU and KS participated in the design of the study and performed the statistical analysis. All authors read and approved the final manuscript.
